# Expression and Significances of Contactin-1 in Human Gastric Cancer

**DOI:** 10.1155/2013/210205

**Published:** 2013-03-31

**Authors:** Ji-Wei Yu, Sheng-Hua Wu, Rui-qi Lu, Ju-gang Wu, Xiao-Chun Ni, Gou-cai Zhou, Hai-guang Jiang, Lin-Hai Zheng, Xiao-Qiang Li, Guang-Ye Du, Bo-Jian Jiang

**Affiliations:** ^1^Department of General Surgery, Shanghai 3rd People's Hospital, School of Medicine, Shanghai Jiao-tong University, Shanghai 201900, China; ^2^Department of Pathology, Shanghai 3rd People's Hospital, School of Medicine, Shanghai Jiao-tong University, Shanghai 201900, China

## Abstract

*Background*. This study aimed at determining the relationship between vascular endothelial growth factor-C (VEGF-C), vascular endothelial growth factor receptor-3 (VEGFR-3), and contactin-1 (CNTN-1) expression in gastric cancer (GC). *Methods*. The expression level of CNTN-1 mRNA and CNTN-1 protein of 33 cases was determined using RT-PCR and Western Blot. And 105 cases were immunohistochemically examined for VEGF-C, VEGFR-3, and CNTN-1 expressions. Assessment of lymphatic vessel density (LVD) was also performed by D2-40 immunostaining. Then we analyzed the relationships between VEGF-C, VEGFR-3, and CNTN-1, as well as their correlations with clinicopathologic features, LVD, and survival time. *Results*. The positivity rate of VEGF-C, VEGFR-3, and CNTN-1 in primary tumor was 56.19%, 64.76%, and 58.09%. The expression of CNTN-1 significantly correlated with VEGF-C (*P* < 0.001) and VEGFR-3 (*P* < 0.001). All of them were closely related to TNM stage, lymphatic invasion, and lymph node involvement (*P* < 0.05). LVD was significantly correlated with VEGF-C (*P* = 0.001), VEGFR-3 (*P* = 0.011), and CNTN-1 expression (*P* < 0.001). VEGF-C, VEGFR-3, and CNTN-1 expression significantly associated with poorer prognosis (*P* < 0.001, *P* = 0.034, *P* = 0.012, resp.). *Conclusion*. CNTN-1 associated with VEGF-C and VEGFR-3 expression in GC. All of them correlated with lymphatic metastasis, which might play an important role in the lymphatic invasion via lymphangiogenesis pathway in GC.

## 1. Introduction

Vascular endothelial growth factor C (VEGF-C) and its receptor, VEGF receptor 3 (VEGFR-3), have been identified as the principal growth factors and the vital receptor for lymphangiogenesis in a variety of human malignancies, including gastric cancer [1–4]. However, the signaling pathway activated by the interaction between VEGF-C and VEGFR-3 in tumor cells is still unknown.

CNTN-1 is a member of the contactin subgroup of the immunoglobulin superfamily which participates in various signal transduction pathways [5]. It associates with two other cell surface proteins which are believed to participate in signal transduction [6, 7]. Recent evidence suggests that the gene for CNTN-1 plays an essential role in tumor invasion and metastasis. Suppression of CNTN-1 expression abolishes the ability of tumor cells to invade Matrigel *in vitro* as well as the polymerization of filamentous-actin and the formation of focal adhesion structures [8]. Knockdown of CNTN-1 results in the extensive inhibition of tumor metastasis and the improvement of survival in an animal model [9], but whether CNTN-1 contributes to the formation of lymphatic network and participates in the process of lymph node metastasis in gastric cancer has not been clarified. There are only few reports that elucidate the relation between CNTN-1 and VEGF-C expression in the growth of primary tumor and lymphatic metastasis in lung adenocarcinoma [8, 9].

The purpose of the present study was to investigate the presence of CNTN-1 in patients with primary gastric cancer using RT-PCR and Western Blot. In addition, we analyzed the relationships between CNTN-1, VEGF-C, and VEGFR-3, and their correlations with clinicopathologic features, LVD, and survival time.

## 2. Methods

### 2.1. Patients and Tumor Specimens

A total of 105 samples were selected from patients who underwent radical gastrectomy (D2 or D3) for primary gastric carcinoma at our hospital, from January 2004 to July 2009. This group of patients included 73 male and 32 female, and the median age of the patients was 62 years old (range 29~82 years old). Among them, fresh tissues of 33 cases from May 2008 to July 2009 were also assessed by RT-PCR for CNTN-1 mRNA and by Western Blot for CNTN-1 protein. None of them accepted any preoperative chemotherapy or radiotherapy. Preoperative informed consent was obtained from each patient included in the study in accordance with institutional guidance. Specimens from primary tumor and normal tissue, identified by pathological observation, at >5 cm distance adjacent to primary lesion were obtained. Samples for immunostaining were fixed in 10% buffered formalin and embedded in paraffin. Pathological and clinical records were reviewed and tumor staging was performed according to the International Union Against Cancer (UICC) classification 5th edition criteria [10]. This study was approved by hospital ethic committee before starting.

### 2.2. RNA Isolation and RT-PCR

Total RNA was extracted from the gastric carcinoma specimens with a RNeasy Mini Kit (Qiagen, CA) according to the manufacturer's instructions. RT-PCR was performed with the isolated RNA, and the primers sequences for CNTN-1 were 5′-TGTTCAGCAAATTCATCCCA-3′ (forward) and 5′-TCTACCCACTCAGGGAATGC-3′ (reverse), and for *β*-actin were 5′-GATGATGATATCGCCGCGCT-3′ (forward) and 5′-TGGGTCATCTTCTCGCGGTT-3′ (reverse). The RT-PCR was performed with extracted RNA and oligomers as templates and primers, respectively. Denaturation was done at 95°C for 10 min. The PCR conditions were 30 cycles of 95°C for 1 min, 55°C for 1 min, and 72°C for 1 min, then 72°C for 10 min. PCR products were visualized by ethidium bromide staining after separation by agarose gel electrophoresis.

### 2.3. Western Blot Analysis

The protein extractions were performed on liquid nitrogen frozen tumor tissues and noncancerous gastric tissues. Protein concentrations were estimated with BCA Protein Assay Kit (Thermo, USA) using bovine serum albumin as the standard. Western Blot was performed as follows: proteins (20 *μ*g) were loaded on a 12% SDS-polyacrylamide gel for electrophoresis. The protein was then transferred to a nitrocellulose membrane. Thereafter, the membranes were blocked with 5% (wt/vol) skimmed milk in tris buffered saline tween 20 (TBST) (50 mM Tris-HCl, 150 mM NaCl, pH = 7.5, 0.1% v/v Tween 20) for 1 h at room temperature, then sequentially incubated in primary antibodies (CNTN-1 rabbit polyclonal antibody, 1 : 1000 dilution or anti-*β*-actin, dilution 1 : 300) overnight at 4°C and HRP-conjugated secondary antibody for 2 h at room temperature. Membranes were washed three times for 5 min each in TBST between antibody incubations. Protein bands were visualized using the BeyoECL Plus (Thermo, USA), with densities determined using a Xuorescence scanner (Bio-Rad, USA).

### 2.4. Immunohistochemistry (IHC)

Serial tissue sections with 4 *μ*m thickness were stained for four endothelial cell markers: VEGF-C goat polyclonal antibody (Abcam, UK), CNTN-1 rabbit polyclonal antibody (Abcam, UK), VEGFR-3 rabbit polyclonal antibody (Abcam, UK), and D2-40 mouse monoclonal antibody (Abcam, UK). Sections were dewaxed and rehydrated by sequential immersion in xylene, graded ethanol, and water. Endogenous peroxidase activity was blocked by 3% hydrogen peroxide for 15 min in methanol. Antigen retrieval was done by heating the slides in microwave oven in 0.01 mmol/L citrate buffer (pH 6.0). After washing in phosphate-buffered saline (PBS), the slides were exposed to 10% normal blocking serum for 10 min to reduce the nonspecific antibody binding, then incubated with the primary antibody, which reacts specifically with VEGF-C (1 : 20 dilution, overnight at 40°C), CNTN-1 (1 : 300 dilution, overnight at 4°C), or VEGFR-3 (1 : 100 dilution, overnight at 4°C) in humid chambers. According to the production instructions, after 3 changes of PBS washing, slides were incubated at 37°C in humid chambers with biotinylated secondary antibody for 30 min. Then incubate sections for 30 min with AB enzyme reagent. Primary antibodies were visualized with peroxidase substrate which had been provided in the kit (ABC staining system of goat, rabbit and mouse, Santa Cruz, USA). Finally, sections were counterstained with hematoxylin. Negative controls were carried out as above by substituting normal serum for the primary antibodies. Sections from previously studied cases of gastric cancer known to positive expression were used as positive controls.

### 2.5. Evaluation of IHC Staining

VEGF-C and CNTN-1 were both observed almost in the cytoplasm of gastric tumor cells. VEGFR-3 was detected not only on tumor cells but also on lymphatic endothelium [11, 12]. Positive reaction was indicated as brown precipitates in these specific locations.

The entire tissue section was scanned to assign the scores. The staining intensity was scored as 0 (negative), 1 (weak), 2 (medium), and 3 (strong). Percentage of staining cells was scored as 0 (0%), 1 (1~25%), 2 (26~50%), 3 (51~75%), and 4 (76~100%), according to the percentages of the positive staining tumor cells in relation to the total tumor cells counted. The percentage of the positive staining cells was calculated as follows: the percentage of the positive staining cells = (the number of positive cells/1000 tumor cells counted) × 100%. The sum of the intensity score and the percentage score was used as the final staining score (0~7). For the purpose of statistical evaluation, tumors having a final staining score of ≥3 were considered to be positive [13]. The final score of 3~5 was classified as low or intermediate expression group, and the final score of 6~7 was assigned as high expression group [14]. All sections were scored by two independent investigators blind to each patient's status under a light microscope, according to both the proportion of stained cells and their intensity.

All of the stained vessels with brown by D2-40 immunostaining were observed as typically positive lymphatic vessel in thin-walled and tube-like structures exhibiting a distinct inner cavity and devoid of red blood cells. LVD (lymphatic vessels density) were determined from the counts of D2-40-positive vessels [12]. D2-40-positive vessel density was assessed by light microscopy of the intratumoral region containing the greatest number of capillaries and small venules (so-called hot spot). Highly vascular areas were identified by scanning tumor sections at low power (×40 and ×100). After the 6 areas of greatest neovascularization were identified, a vessel count was performed at ×200 (field area 0.74 mm^2^), and the mean count of 6 fields was calculated. As in the study of Weidner et al. [15], identification of a lumen was not required for a structure to be considered a blood microvessel. The results were determined independently by two observers.

### 2.6. Statistics

All statistical analyses were performed with the software of SPSS 13.0 (SPSS, Chicago, IL, USA). Statistical analysis of RT-PCR and Western Blot was carried out using Student's *t*-test. The correlations between expression of VEGF-C, CNTN-1, and several clinicopathological parameters were assessed with the chi-squared test as indicated. LVD data were expressed as means ± SD, and statistical analysis was carried out using Student's *t*-test. The Kaplan-Meier method was used to estimate survival as a function of time, and survival differences were analyzed with the log-rank test. The Cox regression model was used for multivariate analysis of prognostic factors. In all of the tests, a *P* value less than 0.05 was considered to be statistically significant.

## 3. Results

### 3.1. Expression of CNTN-1 mRNA and CNTN-1 Protein in Gastric Cancer

**Figure 1:**
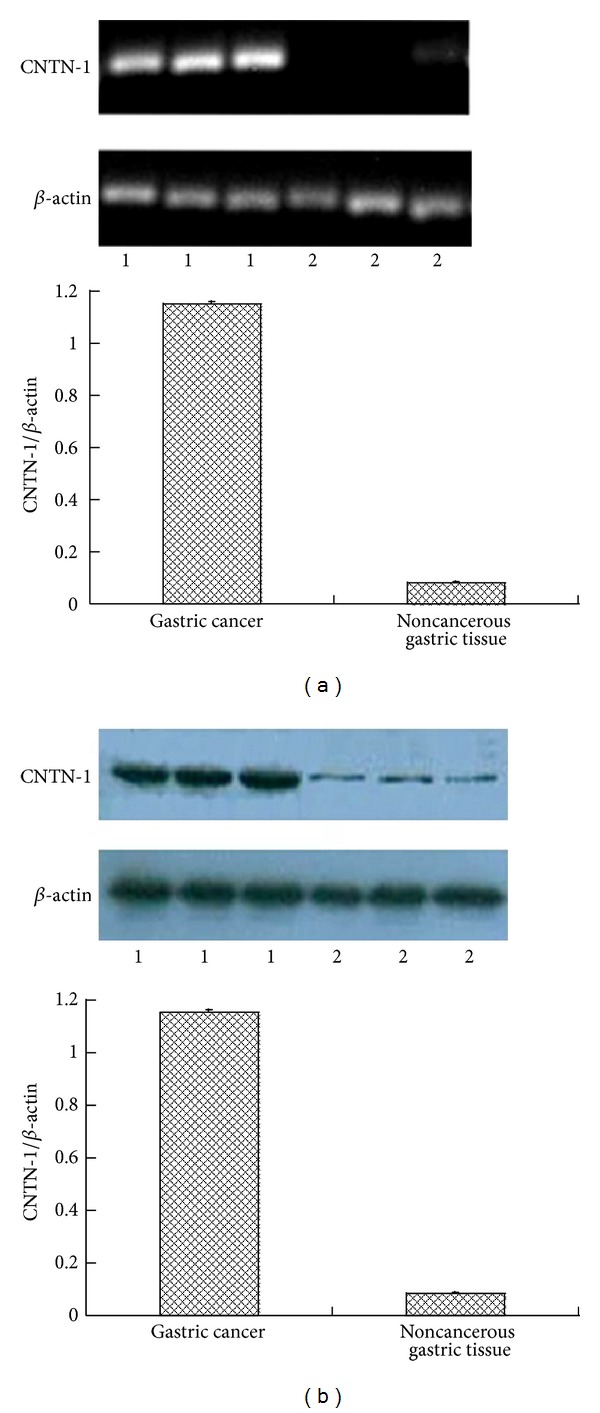
Expression of CNTN-1 mRNA (a) and CNTN-1 protein (b) in gastric cancer. (a) Semiquantitative RT-PCR for expression of CNTN-1 mRNA in gastric cancer. *β*-actin was applied to internal control (1: gastric cancer group; 2: noncancerous gastric tissue group). Tumour samples expressed higher level of CNTN-1 mRNA than that in noncancerous gastric samples (*P* < 0.001). (b) Western Blot for expression of CNTN-1 protein in gastric cancer. *β*-actin was applied to internal control (1: gastric cancer group; 2: noncancerous gastric tissue group). Tumour samples expressed higher level of CNTN-1 protein than that in noncancerous gastric samples (*P* < 0.001).

We initially examined the expression of CNTN-1 mRNAs in gastric cancer by semiquantitative RT-PCR. Different expression level of CNTN-1 mRNA was detected in tumour samples and in noncancerous gastric samples. Tumor samples expressed higher level of CNTN-1 mRNA than that in noncancerous gastric samples. *β*-actin was used as internal control (Figure 1(a)).

CNTN-1 protein expression was analyzed by Western Blot. Different expression level of CNTN-1 protein was detected in tumour samples and in noncancerous gastric samples. Tumor samples expressed higher level of CNTN-1 protein than that in noncancerous gastric samples. *β*-actin was used as internal control (Figure 1(b)).

### 3.2. Expression of VEGF-C, VEGFR-3, and CNTN-1 in Gastric Cancer

**Figure 2:**
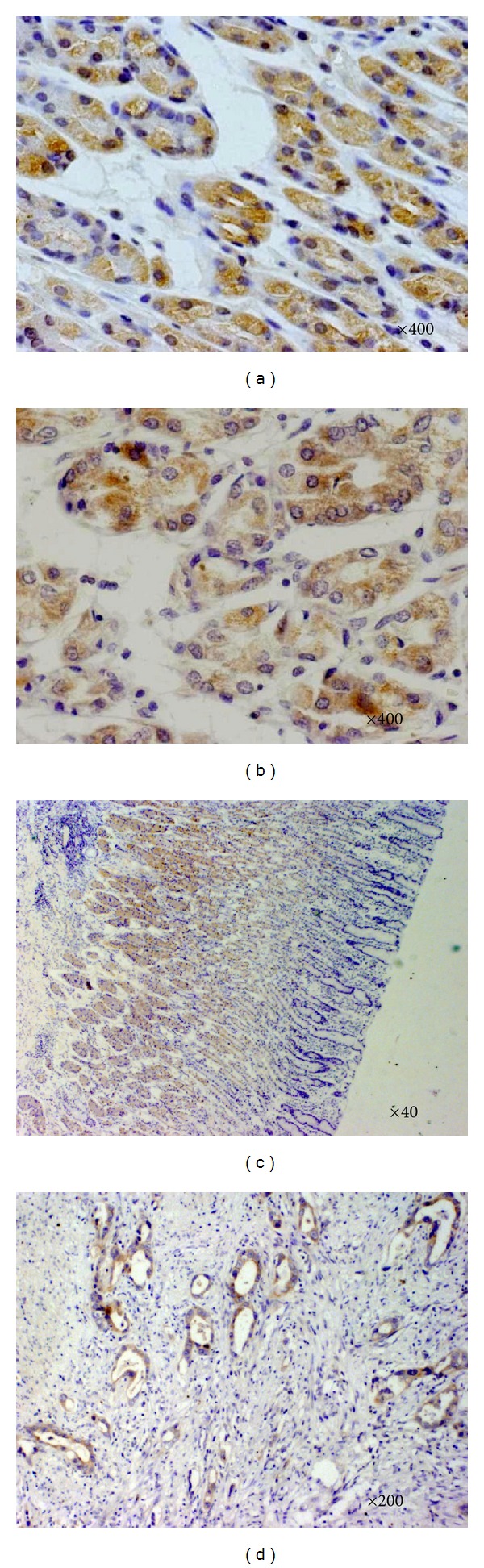
Immunohistochemical labeling for VEGF-C, VEGFR-3, CNTN-1, and D2-40 in primary lesion of gastric carcinoma. (a) VEGF-C expression is indicated as brown precipitates in the cytoplasm in gastric cancer. (b) CNTN-1 expression is observed as a dark brown colour in the cytoplasm. (c) VEGFR-3 expression is observed as a dark brown colour in the cytoplasm. (d) Tumour cells were identified inside D2-40-positive lymphatic vessels in some cases.

VEGF-C, VEGFR-3, and CNTN-1 were widely expressed in the primary lesion of gastric cancer. Both VEGF-C and CNTN-1 were observed almost exclusively in the cytoplasms of gastric tumor cells. The expression of VEGFR-3 was detected in the tumor epithelium and surrounding lymphatic vessels [16]. In the preliminary study, we compared the specificity of antibody against CNTN-1 in gastric ulcer, chronic atrophic gastritis, and gastric cancer. In benign diseases of stomach, no positive staining could be identified in our hand. A few of cells with weakly positive staining were seen in the noncancerous tissue adjacent to primary tumor (Figure 2). The positivity rate of VEGF-C, VEGFR-3, and CNTN-1 in all cases with gastric cancer was 56.19%, 64.76%, and 58.09%, respectively, which was significantly higher than the positive rate of VEGF-C (17.14%), VEGFR-3 (15.23%), and CNTN-1 (21.90%) in control groups (Table 1). Moreover, CNTN-1 positivity was significantly correlated with the presence of VEGF-C (*P* < 0.001) and VEGFR-3 (*P* = 0.001) (Table 2). The Spearman correlation test showed significant correlations among these three proteins expressions.

### 3.3. Correlation of Clinicopathological Parameters with Expression of CNTN-1 and VEGF-C

Expression of VEGF-C, VEGFR-3, and CNTN-1 was all significantly correlated with TNM stage, lymphatic invasion, and lymph node metastasis, but not with age, gender, tumor size, tumor location, Lauren's classification, vascular invasion, or serosa invasion, respectively. Furthermore, the higher metastatic rate of lymph node showed wider expressions of these three proteins. The correlations of VEGF-C, VEGFR-3, and CNTN-1 expressions with clinicopathological parameters are summarized in Table 3. Patients with higher levels of both VEGF-C and CNTN-1 were more likely than those with low or intermediate levels of these proteins to have more advanced stage, more severe lymph node metastasis, lymphatic invasion, and serosa invasion. Most importantly, patients having tumors with higher expression levels of both VEGF-C and CNTN-1 had a shorter survival time of these two groups. The relationships between the level of combined expressions of VEGF-C and CNTN-1 as well as the clinicopathological characteristics of gastric cancer are summarized in Table 4.

### 3.4. Expression of CNTN-1, VEGF-C, and VEGFR-3 Correlated with LVD and Lymphatic Metastasis

D2-40-positively stained vessels were observed typically as thin-walled and tube-like structures exhibiting a distinct inner cavity and devoid of red blood cells. Occasional invasion of the carcinoma cells into the lymph vessels was seen (Figure 2(d)). The average LVD of all cases was (10.26 ± 8.46)/field.

The expression of LVD was significantly higher in patients with vascular invasion, lymphatic invasion, lymph node metastasis, and later TNM stage than that in patients without serosa invasion, lymphatic invasion, lymph node metastasis, and later TNM stage. Furthermore, the higher LVD could be detected in the subgroup of higher lymph node metastatic ratio. This study also revealed the close correlation of VEGF-C, VEGFR-3, or CNTN-1 expression with LVD. The patients with positive expression of VEGF-C, VEGFR-3, or CNTN-1 shared significantly higher LVD than that in the patients with negative expression (Table 5).

### 3.5. Expression of VEGF-C, VEGFR-3, and CNTN-1 Correlated with Survival

**Figure 3:**
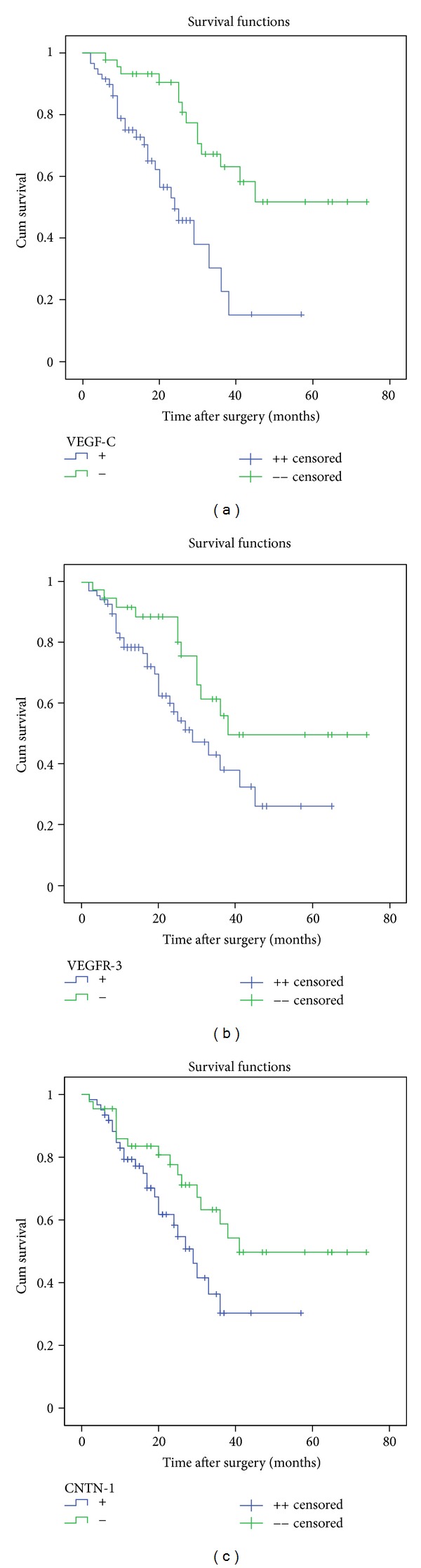
Kaplan-Meier survival curves of patients with gastric cancer according to VEGF-C, VEGFR-3, and CNTN-1 protein expression. Correlation between overall survival of the patients and VEGF-C expression was found to be statistically significant (log rank: *P* < 0.001) as well as that between survival and VEGFR-3 expression (log rank: *P* = 0.034), survival and CNTN-1 expression (log rank: *P* = 0.012).

Within a mean postoperative follow-up duration of 23 ± 16 months (2~74 months), 42 cancer-related deaths occurred: 12 in patients with VEGF-C negative tumors and 30 in the positive group (*χ* ^2^ = 6.603, *P* = 0.010), 9 in patients with VEGFR-3 negative tumors and 33 in the positive group (*χ* ^2^ = 5.850, *P* = 0.016), and 10 in patients with CNTN-1 negative tumors and 32 in the positive group (*χ* ^2^ = 9.415, *P* = 0.002). Kaplan-Meier curves for patients' survival according to the VEGF-C, VEGFR-3, and CNTN-1 status are shown in Figure 3. Patients with VEGF-C-positive, VEGFR-3-positive, and/or CNTN-1-positive tumors had a significant shorter survival time than those with negative tumors. On Cox regression analysis, VEGF-C expression, CNTN-1 expression, lymphatic invasion, and serosa invasion were an independent impact factor on survival, respectively (Table 6).

## 4. Discussion

Recent researches showed that VEGF-C played an important role in the dissemination of many solid tumors [17–19]. By binding to its receptor VEGFR-3, VEGF-C promotes lymphangiogenesis, thus accelerating cancer metastasis to lymph nodes and distant organs [20–22]. Association of VEGF-C with tumor lymphangiogenesis and with lymph node metastasis has been observed in many human carcinomas, including prostate [23], esophageal [24], gastric [25], colorectal [26], cervical cancer [27], and lung cancers [28]. However, neither the signaling pathway activated by the interaction between VEGF-C and VEGFR-3 in epithelial tumor cells nor the biological significance of activation of this axis is understood so far. In the present study, we found that elevated expression of VEGF-C and VEGFR-3 significantly correlated not only with lymphatic invasion and lymph node metastasis but also with the advanced stages of TNM classification. Therefore, the overexpression of VEGF-C is one of many factors involved in the stage of lymph node metastasis.

CNTN-1 is a glycosylphosphatidylinositol (GPI)-anchored 135-kDa cell surface protein that belongs to a family of immunoglobulin (lg) domain-containing cell adhesion molecules (CAMs) that also includes N-CAM, L1, and Nr-CAM [29, 30]. CNTN-1 is located in the 12q11-q12 chromosomal region [5]. It is a member of the contactin subgroup of the immunoglobulin superfamily which participates in various signal transduction pathways [5]. It associates with two other cell surface proteins which are believed to participate in signal transduction. CNTN-1 interacts in transverse with receptor protein tyrosine phosphatase *β* (RPTP*β*) to promote neurite outgrowth [6] and in cis with RPTP*α* [7] to transduce extracellular signals to Fyn kinase, a member of the Src kinase family that regulates cell mobility [31]. The VEGF-C/VEGFR-3 mediated invasion and metastasis of cancer cells were found to require upregulation of CNTN-1 through activation of the Src/p38 MAPK-mediated C/EBP signaling pathway [8]. CNTN-1 was reported expressing in cancers, such as human astrocytic gliomas [32] and lung adenocarcinoma [8].

It is now widely accepted that malignant tumors contain heterogeneous populations of cells of varying metastatic potential [33]. In lung adenocarcinoma cell lines having different capacity of metastasis, genes related to cell adhesion and migration were identified with a customized GEArray and cDNA array containing cDNA sequences corresponding to functions. The gene for CNTN-1 was found to play an essential role in tumor invasion and metastasis [8]. Suppression of CNTN-1 expression abolished the ability of lung adenocarcinoma cells to invade Matrigel *in vitro *as well as the polymerization of filamentous-actin and the formation of focal adhesion structures. Knockdown of CNTN-1 resulted in extensive inhibition of tumor metastasis and improvement of survival in an animal model [9]. Su et al. suggested that CNTN-1 might act as a downstream effector in VEGF-C/VEGFR-3-induced invasion and metastasis via RhoA-mediated mechanisms [8, 9]. Whether CNTN-1 expression has a positive correlation with lymphatic invasion and lymph node metastasis in gastric cancer is still unknown. Our data suggest that CNTN-1 mRNA and CNTN-1 protein is highly expressed in gastric cancer compared to that in noncancerous gastric tissue. Patients with lymphatic invasion, lymph node metastasis, and later TNM staging showed higher expression level of CNTN-1. Association between CNTN-1 expression and clinicopathological features in gastric cancer indicated that CNTN-1 might contribute to the promotion of cancer cell metastasis in primary gastric cancer either. The spearman correlation test showed significant correlations between CNTN-1 and VEGF-C. LVD was significantly higher in the CNTN-1 positive group than that in negative group. VEGF-C, VEGFR-3, and CNTN-1 expression was significantly correlated with the higher LVD values, respectively, indicating the grade of lymphangiogenesis in gastric cancer.

As well known, it had been demonstrated that CNTN-1 might be an adjusting factor in downstream of VEGF-C/VEGFR-3 axis to promote lymph node metastasis. Theoretically, the high expression of VEGF-C occurs unanimously with the high expression of CNTN-1. However, the scores calculated by the sum of positivity intensity and positivity percentage are unavoidable to have some individual deviation, especially as the number of samples is not big enough in this study of ours. On the other hand, for some other proper explanations to this unanimous phenomenon, another adjusting mechanism may exist which should be probed furthermore.

In this study of ours, 26 cases shared the high expressions of both VEGF-C and CNTN-1, and 18 cases shared the high expression of ether VEGF-C or CNTN-1. After comparing the differences in clinicopathological outcomes and prognosis between these two groups, the patients with the high expressions of both VEGF-C and CNTN-1 possessed later TNM stage, easier to result in serosa infiltration, lymphatic vessel invasion, and lymph node metastasis. Much more important, the patients with the high expressions of both VEGF-C and CNTN-1 possessed poorer survival.

We further evaluated the prognostic value of VEGF-C, VEGFR-3, and CNTN-1 expression. Patients with tumors expressing VEGF-C, VEGFR-3, or CNTN-1 had a poorer prognosis as compared with those with expression-negative tumors. On Cox regression analysis, VEGF-C expression, CNTN-1 expression, lymphatic invasion, and serosa invasion were shown to be of statistical significance. A multiple central study with larger samples would be necessary to confirm the prognostic relevance of VEGFR-3, lymph node metastasis, and TNM stage in gastric carcinoma. Our data suggests that the detection of VEGF-C expression, CNTN-1 expression, lymphatic invasion, and serosa invasion may be a useful indicator of poorer prognosis in gastric cancer, respectively.

## 5. Conclusions

The expression of CNTN-1 correlated with the expression of VEGF-C and VEGFR-3. All of them correlated with the presence of lymphatic invasion and prognosis in gastric carcinoma. This phenomenon may raise the possibility that intrinsic relationship of VEGF-C and CNTN-1 overexpression might play an important role in the lymphatic invasion in patients with gastric cancer. Also, the predictive ability for mortality of lymphatic metastasis can be improved with the combined evaluation of the immunohistochemical expression of these three proteins.

## Figures and Tables

**Table 1:** The positive rate comparison between experimental group and control group (*n*/%).

	Carcinoma tissues	Noncancerous gastric tissue	*P* value
VEGF-C			
(+)	59/56.19	18/17.14	0.01
(−)	46/43.81	87/82.86	
VEGFR-3			
(+)	68/64.76	23/15.23	0.01
(−)	37/35.24	82/84.77	
CNTN-1			
(+)	61/58.09	16/21.90	0.01
(−)	44/41.91	89/78.10	

**Table 2:** Correlation of VEGF-C, VEGFR-3, and CNTN-1 expression (*n*).

	VEGFR-3				CNTN-1			
	(+)	(−)	*R* value	*P* value	(+)	(−)	*R* value	*P* value
VEGF-C			0.155	<0.001			0.143	<0.001
(+)	48	11			44	15		
(−)	20	26			17	29		
VEGFR-3							0.118	0.001
(+)	—		—		48	20		
(−)					13	24		

Note: results were calculated by Fisher's exact test.

**Table 3:** Correlation of clinicopathologic parameters with VEGF-C, VEGFR-3, and CNTN-1 expression in gastric cancer (*n*/%).

Clinicopathological features	Total cases	VEGF-C			VEGFR-3			CNTN-1		
		(+)	(−)	*P *	(+)	(−)	*P *	(+)	(−)	*P *
Age (ys)				0.925						0.972
≧60	69/65.7	39/66.1	30/65.2		47/69.1	22/59.5		40/65.6	29/65.9	
<60	36/34.3	20/33.9	16/34.8		21/30.9	16/40.5		21/34.4	15/34.1	
Gender				0.676			0.571			0.545
Male	73/69.5	42/71.2	31/67.4		46/67.6	27/73.0		41/67.2	32/72.7	
Female	32/30.5	17/28.8	15/32.6		22/32.4	10/27.0		20/34.8	12/27.3	
Tumor size				0.177			0.317			0.035
≧5 cm	58/55.2	36/61.0	22/47.8		40/58.8	18/48.6		39/63.9	19/43.2	
<5 cm	47/44.8	23/39.0	24/52.2		28/41.2	19/51.4		22/36.1	25/56.8	
Tumor location				0.218			0.320			0.098
Upper	17/16.2	11/18.6	6/13.0		13/19.1	4/10.8		6/9.8	11/25.0	
Middle	34/32.4	15/25.5	19/41.3		19/27.9	15/40.5		20/32.8	14/31.8	
Lower	54/51.4	33/55.9	21/45.7		36/52.9	18/58.7		35/57.4	19/43.2	
Lauren's classification				0.071			0.885			0.328
Intestinal	70/66.7	35/59.3	35/76.1		45/66.2	25/67.6		43/70.5	27/61.4	
Diffuse	35/33.3	24/40.7	11/23.9		23/33.8	12/32.4		18/29.5	17/38.6	
TNM stage				0.001			<0.001			0.002
I + II	41/39.0	15/25.4	26/56.5		18/26.5	23/62.2		16/26.2	25/56.8	
III + IV	64/61.0	44/74.6	20/43.5		50/73.5	14/37.8		45/73.8	19/43.2	
Vascular invasion				0.526			0.179			0.385
(+)	26/24.8	16/32.2	10/15.2		14/20.6	12/32.4		17/27.9	9/20.5	
(−)	79/75.2	43/67.8	36/84.8		54/79.4	25/67.6		44/72.1	35/79.5	
Serosa invasion				0.363			0.084			0.148
(+)	37/35.2	23/39.0	14/30.4		28/41.2	9/24.3		18/29.5	19/43.2	
(−)	68/64.8	36/61.0	32/69.6		40/58.8	28/75.7		43/70.5	25/56.8	
Lymphatic invasion				<0.001			<0.001			0.001
(+)	66/62.9	49/83.1	17/37.0		54/79.4	12/32.4		47/77.0	19/43.2	
(−)	39/37.1	10/16.9	29/63.0		14/20.6	25/67.6		14/23.0	25/56.8	
Lymph node metastasis				<0.001			<0.001			<0.001
(+)	70/66.7	49/83.1	21/45.7		54/79.4	16/43.2		50/82.0	20/45.5	
(−)	35/33.3	10/16.9	25/54.3		14/20.6	21/56.8		11/18.0	24/54.5	

**Table 4:** Combined expressions of VEGF-C and CNTN-1 associated with clinicopathologic characteristics (*n*/%).

Characteristic	Both high expression^a^ (*n* = 26)	Both non high expression^b^ (*n* = 18)	*P* value
Age (ys)			0.761
≧60	16/61.5	12/66.7	
<60	10/38.5	6/33.3	
Gender			0.323
Male	20/76.9	11/61.1	
Female	6/23.1	7/38.9	
Tumor size			0.234
≧5 cm	17/65.4	8/47.1	
<5 cm	9/34.6	9/52.9	
Tumor location			0.292
Upper	10/38.4	3/16.7	
Middle	8/30.8	7/38.9	
Lower	8/30.8	8/44.4	
Lauren's classification			0.395
Intestinal	14/53.8	12/66.7	
Diffuse	12/46.2	6/33.3	
TNM stage			0.023
I + II	5/19.2	10/55.6	
III + IV	21/80.8	8/44.4	
Vascular invasion			0.325
(+)	15/57.7	13/72.2	
(−)	11/42.3	5/27.8	
Serosa invasion			0.014
(+)	9/34.6	13/72.2	
(−)	17/63.4	5/27.8	
Lymphatic invasion			0.005
(+)	25/96.2	11/61.1	
(−)	1/3.8	7/38.9	
Lymph node metastasis			0.013
(+)	25/96.2	12/66.7	
(−)	1/3.8	6/33.3	
Lymph node metastatic ratio			0.395
≧20%	12/46.2	6/33.3	
<20%	14/53.8	12/66.7	
Average survival (mean ± SD) (months)	16.8 ± 3.8	23.2 ± 5.0	0.032

Note: “a”: indicates that expression of both VEGF-C and CNTN-1 in tumor cells is high. “b”: indicates samples with combined expressions of VEGF-C and CNTN-1 except those of both high expressions.

**Table 5:** Relationship between LVD and other clinicopathological features.

Clinicopathological features	Total cases	LVD (mean ± SD)	*P* value
Age (ys)			
≧60	69	10.94 ± 8.27	0.261
<60	36	8.97 ± 8.79	
Gender			
Male	73	9.71 ± 8.25	0.314
Female	32	11.52 ± 8.92	
Tumor size			
≧5 cm	58	11.05 ± 8.35	0.291
<5	47	9.29 ± 8.59	
Tumor location			
Upper	17	11.62 ± 7.64	0.435
Middle	34	8.78 ± 8.41	
Lower	54	10.77 ± 8.75	
Lauren's classification			
Intestinal	70	10.21 ± 8.51	0.934
Diffuse	35	10.36 ± 8.49	
TNM stage			
I + II	41	7.41 ± 9.08	0.008
III + IV	64	12.09 ± 7.56	
Vascular invasion			
(+)	26	13.92 ± 6.70	0.004
(−)	79	9.06 ± 8.67	
Serosa invasion			
(+)	37	11.5 ± 7.82	0.257
(−)	68	9.59 ± 8.77	
Lymphatic invasion			
(+)	66	12.83 ± 7.46	<0.001
(−)	39	5.92 ± 8.37	
Lymph node metastasis			
(+)	70	12.20 ± 7.87	0.001
(−)	35	6.39 ± 8.37	
Lymph node metastatic ratio			
≧20%	51	13.40 ± 7.63	<0.001
<20%	54	7.30 ± 8.19	
VEGF-C			
(+)	59	12.63 ± 7.41	0.001
(−)	46	7.23 ± 8.34	
VEGFR-3			
(+)	68	13.11 ± 7.01	0.011
(−)	37	9.12 ± 8.13	
CNTN-1			
(+)	61	12.80 ± 7.95	<0.001
(−)	44	6.75 ± 7.96	

**Table 6:** Multivariate survival analysis (Cox proportional hazards model).

Variables	Coefficient *β*	Standard error	Chi-squared	*P* value	Relative risk ratio	95% Confidence interval
VEGF-C expression	0.907	0.377	5.795	0.016	0.404	0.193~0.845
VEGFR-3 expression	0.207	0.383	0.291	0.590	1.230	0.580~2.605
CNTN-1 expression	0.812	0.382	4.524	0.033	0.444	0.210~0.938
Lymphatic invasion	1.389	0.614	5.117	0.024	0.249	0.075~0.831
Lymph node metastasis	0.264	0.580	0.207	0.649	1.302	0.418~4.057
Serosa invasion	1.245	0.421	8.732	0.003	0.288	0.126~0.658
TNM stage	0.426	0.491	0.753	0.385	0.653	0.250~1.709
